# Treating Hepatitis C Virus Infection in Jails as an Offset to Declines in Treatment Activity in the Community, New York City, NY, 2014–2020

**DOI:** 10.1016/j.focus.2024.100185

**Published:** 2024-01-06

**Authors:** Justin Chan, Matthew J. Akiyama, Emily Julian, Rodrigue Joseph, Wendy McGahee, Zachary Rosner, Patricia Yang, Ross MacDonald

**Affiliations:** 1 NYC Health + Hospitals/Bellevue, New York, New York; 2 Department of Medicine, NYU Grossman School of Medicine, New York, New York; 3 NYC Health + Hospitals/Correctional Health Services, NYC Health + Hospitals, New York, New York; 4 Montefiore Einstein Department of Medicine, Albert Einstein College of Medicine, Bronx, New York; 5 NYC Health + Hospitals/Woodhull, NYC Health + Hospitals, New York, New York

**Keywords:** Hepatitis C, direct-acting antiviral, jails

## Abstract

**Introduction:**

There are scant data on implementation of large-scale direct-acting antiviral treatment for hepatitis C virus in jails in the U.S. New York City Health + Hospitals/Correctional Health Services aimed to scale up hepatitis C virus treatment in the New York City jail system. This study describes the trends in annual hepatitis C virus treatment in New York City jails compared with those in Medicaid-funded treatment in the New York City community from 2014 to 2020.

**Methods:**

In this observational study, we extracted annual counts of direct-acting antiviral prescriptions for hepatitis C virus for those (1) in the New York City community who were covered by Medicaid and (2) those detained in New York City jails for 2014–2020. Data sources were New York City Department of Health and Mental Hygiene annual reports and Correctional Health Services treatment records, respectively. We used linear regression analysis to test for significant trends in annual treatment in these 2 cohorts during 2015–2019.

**Results:**

From 2015 to 2019, treatments started in New York City jails increased annually (*p*=0.001), whereas Medicaid-funded prescriptions in the New York City community declined since a peak in 2015 (*p*<0.001). In 2019, New York City jail-based treatment initiations totaled the equivalent of 10% of treatment covered by Medicaid in New York City, up from 0.3% in 2015.

**Conclusions:**

Scale up of jail-based hepatitis C virus treatment is an important strategy to offset declines observed in the community. Addressing barriers to care in jail, such as improving testing, linkage to care, and affordability of direct-acting antivirals for jail-based health services, can help sustain high levels of treatment in U.S. jails and other carceral facilities.

## HIGHLIGHTS

• Annual hepatitis C virus (HCV) treatment started in New York City jails increased significantly from 2015 to 2019.
• Annual HCV treatment in the New York City community has declined significantly from 2015 to 2019.
• Scaling up HCV treatment in jails is critical to achieving national HCV elimination.

## GRAPHICAL ABSTRACT

**Figure:**
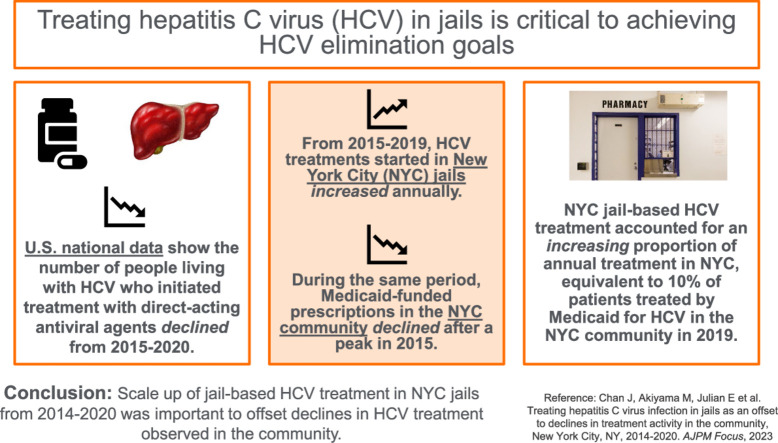

## INTRODUCTION

With a fragmented healthcare system and insurance-related barriers to treatment, the U.S. is not on track for the WHO's 2030 hepatitis C virus (HCV) elimination targets.^1^ The 2021 HHS Viral Hepatitis National Strategic Plan highlights several populations, including people detained in carceral facilities, that need prioritization to improve HCV testing and treatment.^2^ The fiscal year 2024 Biden–Harris administration budget proposal includes a 5-year plan to advance HCV elimination in the U.S.^3^ Despite a high prevalence of HCV-infected individuals in U.S. carceral facilities, scant data have been published regarding implementation of large-scale HCV direct-acting antiviral (DAA) treatment programs in U.S. jails.

Jails in the U.S. are an important setting for delivering HCV treatment. In contrast to prisons, jails are primarily short-stay remand facilities with high turnover. U.S. jails had 10.3 million admissions in 2019, compared with 576,956 at federal and state prisons.^4^ Many HCV-viremic individuals are incarcerated in the New York City (NYC) jail system, with at least 4,665 admitted during 2014–2017,^5^ approximately 5% of the estimated 91,000 people in NYC living with chronic HCV, defined by evidence of HCV viremia.^6^ NYC Health + Hospitals/Correctional Health Services (CHS) provides health care for persons incarcerated in NYC jails, which had an average daily population of 7,400 in 2019. CHS is a division of NYC Health + Hospitals, the nation's largest municipal public healthcare system. Many who enter the jail system experience barriers to HCV treatment in the community.^7^ As such, CHS leverages the jail health setting as a key point of intervention to treat people living with HCV.

From 2016 onward, CHS made concerted efforts to scale up HCV treatment in jail. CHS negotiated preferred pricing with pharmaceutical partners, which in turn helped secure more local funding by improving the underlying cost–benefit balance of expanding HCV treatment. CHS also recruited more staff to support and expand treatment, including prescribers, care coordinators, and patient educators. The goal of this study was to evaluate the outcome of these efforts to scale up treatment in jail in the context of treatment activity trends in the surrounding NYC community.

## METHODS

### Study Population

This observational study included all patients from 2014 to 2020 who were eligible for and prescribed DAA therapy for HCV (1) while in the NYC community and covered by Medicaid insurance and (2) while detained in NYC jails. New York State Medicaid covers DAA therapy for people who have active HCV, defined as those who have a positive HCV RNA test. There are no fibrosis restrictions, substance use restrictions, or prescriber restrictions. Retreatment authorization requires confirmation of patient readiness and adherence along with consideration of potential future drug or alcohol use.^8^ CHS offers HCV screening to all individuals on admission to jail. Patients with HCV viremia are referred to a CHS physician for further evaluation. Treatment eligibility is based on the American Association for the Study of Liver Diseases/Infectious Diseases Society of America HCV Guidelines.^9^ NYC jails averaged 7,400 persons incarcerated on a given day in 2019 compared with 3.39 million Medicaid enrollees in NYC.^10^

### Measures

We tallied the number of patients treated each year in each group using (1) data published by the NYC Department of Health and Mental Hygiene^6^ for Medicaid-funded treatment in NYC and (2) the CHS HCV treatment database that captures all treatment in NYC jails. Treatments in jail were stratified by those who started treatment in the community prior to incarceration and those who started treatment while in jail. Patients who started treatment in NYC under Medicaid coverage prior to incarceration are captured in the Medicaid-funded community treatment cohort. If they get incarcerated mid-treatment, CHS continues the treatment that was started in the community. Those individuals are not counted as treatment started while in jail. The jail cohort for this analysis only includes patients who started a new course of HCV treatment in jail.

### Statistical Analysis

Over the study time period, we compared total annual treatment courses funded by Medicaid in NYC with treatment courses started in NYC jails by CHS by absolute numbers and percentages. We used simple and multiple linear regression to examine the trends in annual treatment initiation counts in the community and in jail.

IRB approval was not obtained. These data were collected for public health surveillance activities or for routine programmatic monitoring. This study did not meet the criteria for human subjects research.

## RESULTS

**Figure 1:**
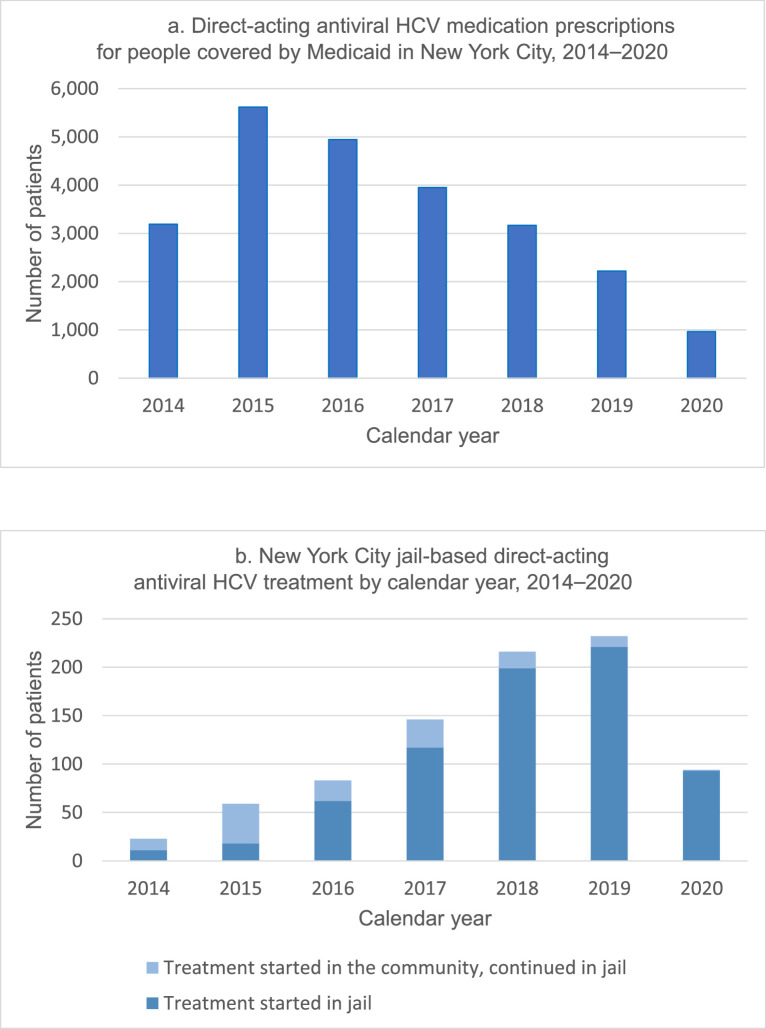
Annual number of people prescribed direct-acting antiviral HCV treatment (A) among people covered by Medicaid in New York City from 2014 to 2020, and (B) among people detained in New York City jails from 2014 to 2020. Source: New York City Department of Health and Mental Hygiene.^6^ The tallies for Medicaid-funded treatment do not include treatment in jail because Medicaid coverage is suspended for those who are incarcerated. HCV, hepatitis C virus.

DAA treatment for HCV has been offered by CHS to people detained in NYC jails since 2014. Figure 1A and B shows annual HCV treatment from 2014 to 2020 in the NYC community and jails, respectively. From 2015 to 2019, there was a statistically significant annual decline in DAA prescriptions covered by Medicaid in the NYC community, whereas there was a statistically significant annual increase in annual DAA prescriptions started in NYC jails. We fit a linear model to test the interaction between the site of HCV treatment initiation and time (years 2015–2019). The interaction was statistically significant with *p*<0.001. We then fit linear models for the data of each initiation site. The slope of the regression line for community-based treatment over time was negative and different from 0, with *p*<0.001. The slope of the regression line for jail-initiated treatment over time was positive and different from 0, with *p*=0.001. The years 2014 and 2020 were excluded from the trend analysis. The year 2014 was the first year after interferon-sparing DAA regimens were first approved by the Food and Drug Administration, and therefore DAA therapy was not widely available. The year 2020 was excluded because the coronavirus disease 2019 (COVID-19) pandemic caused major disruptions in access to various medical services, including HCV testing and treatment.

In 2019, CHS initiated treatment for 221 incarcerated patients, representing the equivalent of approximately 10% of patients treated by Medicaid for HCV in NYC, up from 0.3% in 2015.^6^ One patient received 2 separate HCV treatment courses from CHS in jail in 2018 owing to reinfection. The rest of the treatment courses initiated in jail were prescribed to unique patients. Community and jail treatment totals more than halved in 2020 in the face of the COVID-19 pandemic, but CHS still started 93 patients on treatment while in jail that year. In comparison, HCV DAA prescriptions in the NYC community for people covered by Medicaid have fallen annually since 2015,^6^ suggesting that community-based treatment may be declining, whereas jail-initiated treatment increased. Medicaid tallies do not include treatment in jail because Medicaid coverage is suspended for those who are incarcerated.

## DISCUSSION

The CHS HCV treatment program demonstrates that it is feasible and important to scale up DAA treatment in a large urban jail even when treatment in the surrounding community may have been declining. Further efforts should be taken to increase treatment in jails because it appears difficult to maintain high levels of treatment in the community since a peak in 2015. The declining trend in NYC community-based treatment in our analysis mirrors the pattern observed nationally from 2015 to 2020 on the basis of national pharmacy claims data.^11^ Moreover, scaling up HCV screening and treatment in correctional facilities is cost-effective and would reduce HCV transmission, morbidity, and mortality in the surrounding community.^12^ Barriers to HCV treatment in jails and surrounding communities, respectively, may differ and require tailored solutions. We observed a significant increase in annual HCV treatment in the NYC jail system over time from 2015 to 2019, with a decrease in 2020 due to the COVID-19 pandemic response. During the early phase of the pandemic, some nonurgent routine medical services in the jails, including HCV treatment initiation, were temporarily suspended owing to widespread COVID-19 outbreaks in many areas of the jail system.^13^ Once the outbreaks were better controlled, all routine medical services, including HCV treatment, resumed.

HCV treatment in jail is highly effective. Although one concern raised about starting treatment in jail is whether short lengths of stay may negatively affect treatment outcomes, our other analysis based on available virologic data found an 88% sustained virologic response rate at 12 weeks among an NYC jail treatment cohort.^14^ However, those discharged to the community mid-treatment were at higher risk of not achieving virologic response at 12 weeks than those who completed treatment in jail,^14^ highlighting the importance of improved support during transitions of care to ensure treatment completion.

Although CHS was successful at scaling up HCV treatment in 2014–2019, our other analysis of the cascade of care found that only 5% of HCV-viremic patients started treatment while in jail.^5^ To allow treatment of more patients, it is critical to alleviate barriers to accessing HCV treatment while in jail, such as delays in treatment initiation, and barriers after return to the community, such as lack of insurance coverage, competing priorities, and active substance use.^15^

CHS is well positioned among peer jail systems to deliver large-scale HCV treatment because it is not for profit and enjoys municipal budget support for HCV medication. This may be challenging to replicate at scale nationally without addressing barriers to care. Solutions need to be developed to increase HCV treatment in the correctional system given the concerning declines in community-based treatment. These solutions may come through research and quality improvement efforts to establish more efficient models of care, such as nurse-led treatment,^16^ or advocacy for laws to allow states to cover incarcerated people under Medicaid.^17^ Medicaid coverage during incarceration may improve access to HCV treatment, instead of relying on disparate and often limited local budgets for healthcare services at correctional facilities. Medicaid coverage may also facilitate transitions of care from jail to the community because one reason for interruptions in HCV treatment courses is delays or challenges in reinstating Medicaid coverage upon return to the community.

Several additional challenges must be addressed to expand jail-based treatment programs nationally. Although public health guidelines^18^ now recommend routine opt-out HCV testing in jails, few carceral facilities offer routine testing.^19^ Given the rapid turnover of patients in jail systems, incorporating accurate point-of-care HCV viral load testing^20^ could improve the efficiency of diagnosing active infection and potentially facilitate earlier initiation of treatment during a jail stay. Although Medicaid coverage remains suspended during incarceration, DAA drug prices in the U.S. are expensive for many jail systems that rely on local funding. One solution may be leveraging purchasing coalitions for flat-fee subscription models.^3^^,^^21^ With short stays, jail-based testing and treatment programs need to be accompanied by robust linkage to community care to avoid treatment interruption upon return to the community. Peer navigation starting during incarceration and continuing through return to the community has demonstrated effectiveness in sustaining HIV viral suppression.^22^ These services should be further studied to evaluate effectiveness in improving HCV linkage to care after incarceration.

Many barriers to treatment exist in the community because the most motivated community-dwelling patients were likely treated soon after DAA therapy first became available, and those who are now still living with HCV may be unaware of their diagnosis, experience other barriers to HCV care, or have competing priorities. Until recently, screening guidance focused on risk factor– and birth cohort–based screening, resulting in some of the untreated population remaining undiagnosed. Among those aware of their infection, remaining barriers to care may include restrictions on DAA prescriptions by insurance plans and low rates of linkage to care, especially for those with mental illness and substance use disorders.^7^

People who use drugs (PWUD) account for a large proportion of HCV cases, but they also experience barriers to care such as underdiagnosis and challenges with linkage to care in the community.^23^ This study found that PWUD who had interactions with police were more likely to have prior HCV testing. Therefore, although access to HCV care in the community may be challenging for PWUD for various reasons, those who have interaction with the criminal justice system may be presented with opportunities for HCV testing and treatment. This is consistent with our observation of an increasing trajectory in HCV treatment in NYC jails, relative to the decrease in the community.

Given the high prevalence of HCV relative to that in the general community, carceral health systems and particularly jail settings are key points of intervention to treat the hardest-to-reach populations. Other jurisdictions such as Australia have also found a significant proportion of overall HCV treatment initiation occurring in prisons, ranging from 10% to 73% of the jurisdictional totals in 2021.^24^ Most incarcerated individuals will return to the community eventually, so we expect benefits of HCV treatment in carceral settings to accrue largely in the community, including prevention of further HCV transmission and reduction of decompensated cirrhosis, hepatocellular carcinoma, and liver transplantation. The continued scaling up of HCV treatment in the NYC jail system since 2015 demonstrates that jail-based treatment can help mitigate declines in treatment activity in jurisdictions such as NYC, but jail-specific barriers to treatment should be reduced.

### Limitations

Medicaid-funded treatment does not capture all HCV treatment activity in the community. However, Medicaid is a U.S. federal/state/local program and the largest source of funding for medical services for people with limited income in the community. Therefore, Medicaid prescribing offers an important indication of community-based treatment trends. In 2019, approximately 40% of NYC residents were enrolled in this public benefit,^10^ one of the least restrictive for HCV DAA prescriptions among other states.^25^ Our data are consistent with the pattern of HCV treatment initiation declining nationally since 2016.^26^

We do not have data available to describe the demographics, clinical characteristics, and clinical outcomes of the NYC community covered by Medicaid insurance, so we are unable to characterize differences between that cohort and the cohort treated in jail. Although the characteristics may differ between the 2 cohorts, our conclusions are still valid on the basis of the observed differences in treatment activity trends. We do not have data available to quantify the number of prescriptions covered by Medicaid that were retreatments for the same patient (i.e., for reinfection or treatment failure). However, if retreatments occurred and were captured as separate prescriptions, our data would represent an overcount of the number of individuals treated in the community and result in an underestimation of our conclusions.

It is possible that a small proportion of the jail-based cohort started treatment in jail, got discharged to the community mid-treatment, and continued treatment through a Medicaid-funded prescription. This would lead to the individual being counted in both the jail cohort and the community-based Medicaid cohort, but this would only attenuate the contrasting trends in annual community- versus jail-based treatment over time and cause an underestimation of the conclusions we are drawing (i.e., that community-based treatment is declining, whereas jail-based treatment is increasing over time). Conversely, an individual who started treatment in the community under Medicaid and continued treatment in jail would only be counted in the community-based cohort. The jail treatment cohort only included those who started a new treatment course in jail, which was not covered by Medicaid.

## CONCLUSIONS

Declines in HCV treatment activity in U.S. communities such as NYC are especially concerning because the U.S. is not on track to achieve HCV elimination targets.^1^^,^^11^^,^^26^ The NYC jail experience demonstrates that increasing HCV treatment in jail is achievable and will be important to continue to help offset declines in the surrounding community. Barriers to treatment may be different during and after incarceration, respectively, and each requires tailored solutions.^15^ Further investment related to the proposal to eliminate HCV in the U.S.^3^ should increase treatment in nontraditional healthcare settings, such as carceral facilities, substance use treatment centers, and homeless shelters. Among carceral health settings, jails are uniquely positioned to reach many persons among underserved populations living with HCV.^5^^,^^14^ Achieving HCV elimination goals will require sustained investment to effectively scale up HCV treatment and prevention programs in jails, especially given the declines in treatment observed in the surrounding community.

## CRediT authorship contribution statement

**Justin Chan:** Conceptualization, Data curation, Formal analysis, Investigation, Methodology, Visualization, Writing – original draft. **Matthew J. Akiyama:** Formal analysis, Writing – review & editing. **Emily Julian:** Formal analysis, Writing – review & editing. **Rodrigue Joseph:** Formal analysis, Writing – review & editing. **Wendy McGahee:** Formal analysis, Writing – review & editing. **Zachary Rosner:** Formal analysis, Writing – review & editing. **Patricia Yang:** Supervision, Writing – review & editing. **Ross MacDonald:** Conceptualization, Supervision, Writing – review & editing.

## References

[1] Razavi H, Sanchez Gonzalez YS, Yuen C, Cornberg M (2020). Global timing of hepatitis C virus elimination in high-income countries. Liver Int.

[2] HHS (2020). https://www.hhs.gov/sites/default/files/Viral-Hepatitis-National-Strategic-Plan-2021-2025.pdf.

[3] Fleurence RL, Collins FS (2023). A national hepatitis C elimination program in the United States: a historic opportunity. JAMA.

[4] Total correctional population. Bureau of Justice Statistics. https://www.bjs.gov/index.cfm?ty=tp&tid=11. Updated 2021. Accessed February 14, 2021.

[5] Chan J, Kaba F, Schwartz J (2020). The hepatitis C virus care cascade in the New York City jail system during the direct acting antiviral treatment era, 2014–2017. EClinicalmedicine.

[6] New York City Department of Health and Mental Hygiene (2022). https://www.nyc.gov/assets/doh/downloads/pdf/cd/hepatitis-abc-annual-report-2021.pdf.

[7] Jain MK, Thamer M, Therapondos G (2019). Has access to hepatitis C virus therapy changed for patients with mental health or substance use disorders in the direct-acting-antiviral period?. Hepatology.

[8] Center for Health Law and Policy Innovation of Harvard Law School, National Viral Hepatitis Roundtable (2023). https://stateofhepc.org/states/new-york/.

[9] HCV guidance: recommendations for testing, managing, and treating hepatitis C. AASLD/IDSA. https://www.hcvguidelines.org/. Accessed September 16, 2023.

[10] New York State Department of Health (2019). https://www.health.ny.gov/health_care/medicaid/regulations/global_cap/monthly/sfy_2018-2019/docs/mar_2019_report.pdf.

[11] New estimates reveal declines in hepatitis C treatment in the U.S. between 2015 and 2020. Centers for Disease Control and Prevention. https://www.cdc.gov/nchhstp/newsroom/2021/2014-2020-hepatitis-c-treatment-estimates.html. Updated 2021. Accessed September 19, 2023.

[12] He T, Li K, Roberts MS (2016). Prevention of hepatitis C by screening and treatment in U.S. prisons. Ann Intern Med.

[13] Chan J, Burke K, Bedard R (2021). COVID-19 in the New York City jail system: epidemiology and health care response, March–April 2020. Public Health Rep.

[14] Chan J, Schwartz J, Kaba F (2020). Outcomes of hepatitis C virus treatment in in the New York City jail population: successes and challenges facing scale-up of care. Open Forum Infect Dis.

[15] Kamat S, Kondapalli S, Syed S (2023). Access to hepatitis C treatment during and after incarceration in New Jersey, United States: a qualitative study. Life (Basel).

[16] Overton K, Clegg J, Pekin F (2019). Outcomes of a nurse-led model of care for hepatitis C assessment and treatment with direct-acting antivirals in the custodial setting. Int J Drug Policy.

[17] H.R.6764 – Human Correctional Health Care Act. 115th Congress (2017-2018). https://www.congress.gov/bill/115th-congress/house-bill/6764?s=1&r=31. Accessed September 16, 2023.

[18] CDC recommendations for correctional and detention settings. Centers for Disease Control and Prevention. https://www.cdc.gov/correctionalhealth/rec-guide.html#recommended-actions. Updated 2023. Accessed September 16, 2023.

[19] Maner M, Omori M, Brinkley-Rubinstein L, Beckwith CG, Nowotny K. (2022). Infectious disease surveillance in U.S. jails: findings from a national survey. PLoS One.

[20] Lamoury FMJ, Bajis S, Hajarizadeh B (2018). Evaluation of the Xpert HCV viral load finger-stick point-of-care assay. J Infect Dis.

[21] Trusheim MR, Cassidy WM, Bach PB. (2018). Alternative state-level financing for hepatitis C treatment-the “Netflix Model. JAMA.

[22] Cunningham WE, Weiss RE, Nakazono T (2018). Effectiveness of a peer navigation intervention to sustain viral suppression among HIV-positive men and transgender women released from jail: the LINK LA randomized clinical trial. JAMA Intern Med.

[23] Ozga JE, Syvertsen JL, Pollini RA. (2022). Hepatitis C antibody prevalence, correlates and barriers to care among people who inject drugs in Central California. J Viral Hepat.

[24] Burnet Institute (2022). https://www.burnet.edu.au/knowledge-and-media/research-reports-plus-policy-briefs/australia-s-progress-towards-hepatitis-c-elimination-annual-report-2022/.

[25] Hepatitis C State of Medicaid Access. National Viral Hepatitis Roundtable. http://stateofhepc.org. Updated 2024. Accessed January 18, 2024.

[26] Thomas DL. (2020). State of the hepatitis C virus care cascade. Clin Liver Dis (Hoboken).

